# GERI-VET: A PROGRAM FOR OLDER VETERANS SEEN IN THE EMERGENCY ROOM

**DOI:** 10.1093/geroni/igz038.525

**Published:** 2019-11-08

**Authors:** Denise M Kresevic, Albert Lee, Mustafa Ascha, Todd Smith

**Affiliations:** 1 Northeast Ohio VAMC, Cleveland, Ohio, United States; 2 Northeast Ohio VA Health System, Cleveland, Ohio, United States

## Abstract

Older adults made over 21 million Emergency Room (ER) visits accounting for nearly 46% of all ER hospital admissions in 2015. The ER setting provides not only a unique opportunity to assess patients’ health, functional status and social issues, but also provide recommendations to help coordinate care. Geriatric ER assessments have been associated with reduced avoidable hospitalizations, functional decline, and institutionalization. However, few ER clinicians including physicians, nurses and technicians have received adequate training to perform geriatric screenings and implement timely referrals. In 2014 American College of Emergency physicians and American Geriatric Society published guidelines for care. Based on these guidelines A” Geri-Vet Bootcamp” Program was developed and piloted at the Northeast Ohio VAMC. This program included: simulation emphasizing standardized screenings, and the use of decision support aides for management and referrals for older adults seen in the ER. Following this multi-modal education program, 91% clinicians reported greater ability to apply knowledge learned, 82% clinicians were able to more accurately identify geriatric syndromes, and 86% were able to identify additional resources. Of the patients screened over one year, 73% of patients were identified as being at high risk for falls, 32% had high family caregiver burden, 15% had moderate to severe dementia, and 14% had positive delirium screens. Those veterans screened by Geri Vet trained Staff received significantly more referrals than usual care staff, home care 28.7% vs.15.6%, geriatric clinic 20.5% vs. 11.7% and caregiver support 5.0% vs. 1.3%. Data show hospital admissions have decreased 5-7%. Education and dissemination continues

